# An Uncommon Presentation of the Common Sinusitis

**DOI:** 10.7759/cureus.9263

**Published:** 2020-07-18

**Authors:** Massud Atta, Sidhartha R Ramlatchan, Latha Ganti, Gerald T Delk, John Shivdat

**Affiliations:** 1 Emergency Medicine, Coliseum Medical Centers, Macon, USA; 2 Emergency Medicine, Drexel University, Philadelphia, USA; 3 Emergency Medicine, Envision Physician Services, Nashville, USA; 4 Emergency Medicine, University of Central Florida College of Medicine/Hospital Corporation of America Graduate Medical Education Consortium of Greater Orlando, Orlando, USA; 5 Emergency Medical Services, Polk County Fire Rescue, Bartow, USA; 6 Emergency Medicine, Mercer University School of Medicine, Macon, USA

**Keywords:** pansinusitis

## Abstract

A nine-year-old child was brought to the emergency room by her mother because of an upper respiratory infection symptoms and forehead swelling. The patient was seen by the emergency department physician and diagnosed with an upper respiratory infection; the forehead swelling was felt to be related to forceful coughing. The patient and patient’s mother returned on a second visit because the forehead swelling had not improved. A CT scan of the head was subsequently done which demonstrated pansinusitis.

## Introduction

Sinusitis is one of the most common infections in children and adults. It is a mucosal inflammation of the sinuses and paranasal sinuses lasting up to three to four weeks [1]. Signs and symptoms of sinusitis include headache, facial pain or tenderness, referred pain in the ears and teeth, fever, discolored nasal or postnasal drainage, nasal congestion, sore throat, cough, and occasionally facial swelling [2]. Risk factors include nasal polyps, hay fever or seasonal allergies, asthma, deviated septum, cystic fibrosis, HIV, and other immunocompromising diseases [1]. A common risk factor is having a prolonged upper respiratory infection (URI). Pediatric patients, who develop URIs almost six times per year, are at a higher risk of developing sinusitis [3]. Sinusitis can be caused by infection, allergies, and chemical or particulate irritation of the sinuses [2]. Viral etiologies account for >90% of URIs and do not require treatment with antibiotics [4].

## Case presentation

A nine-year-old girl, with a past medical history of asthma, presented to the emergency department (ED) with her mother after developing URI symptoms associated with a protrusion on her forehead. On the first visit, the ED physician diagnosed her with a URI with etiology being most likely viral, but because the symptoms lasted a couple of weeks, the patient was provided trimethoprim/sulfamethoxazole (TMP-SMX) and prednisolone. The patient continued to take the antibiotics and noticed her URI symptoms subsided, but the protrusion in her forehead did not. So she returned for re-evaluation. 

On the second visit, the patient’s vitals were stable, with a temperature of 98.3°F, heart rate of 89 bpm, oxygen saturation of 99% on room air, and a respiratory rate of 19 breaths per minute. The patient was awake, alert, in no acute distress, and was well developed and appearing. The patient’s head had a large cystic-like protrusion in the mid forehead. She was neurologically intact, without motor or sensory deficits, cranial nerves 2-12 were intact, and reflexes were equal bilaterally with no cerebellar deficits. Eyes showed no nystagmus, normal conjunctiva, normal visual acuity, and no scleral icterus. Her neck was supple, without signs of meningismus or adenopathy. Breath sounds were normal bilaterally, and heart sounds were normal without murmurs, rubs, or gallops.

**Figure 1:**
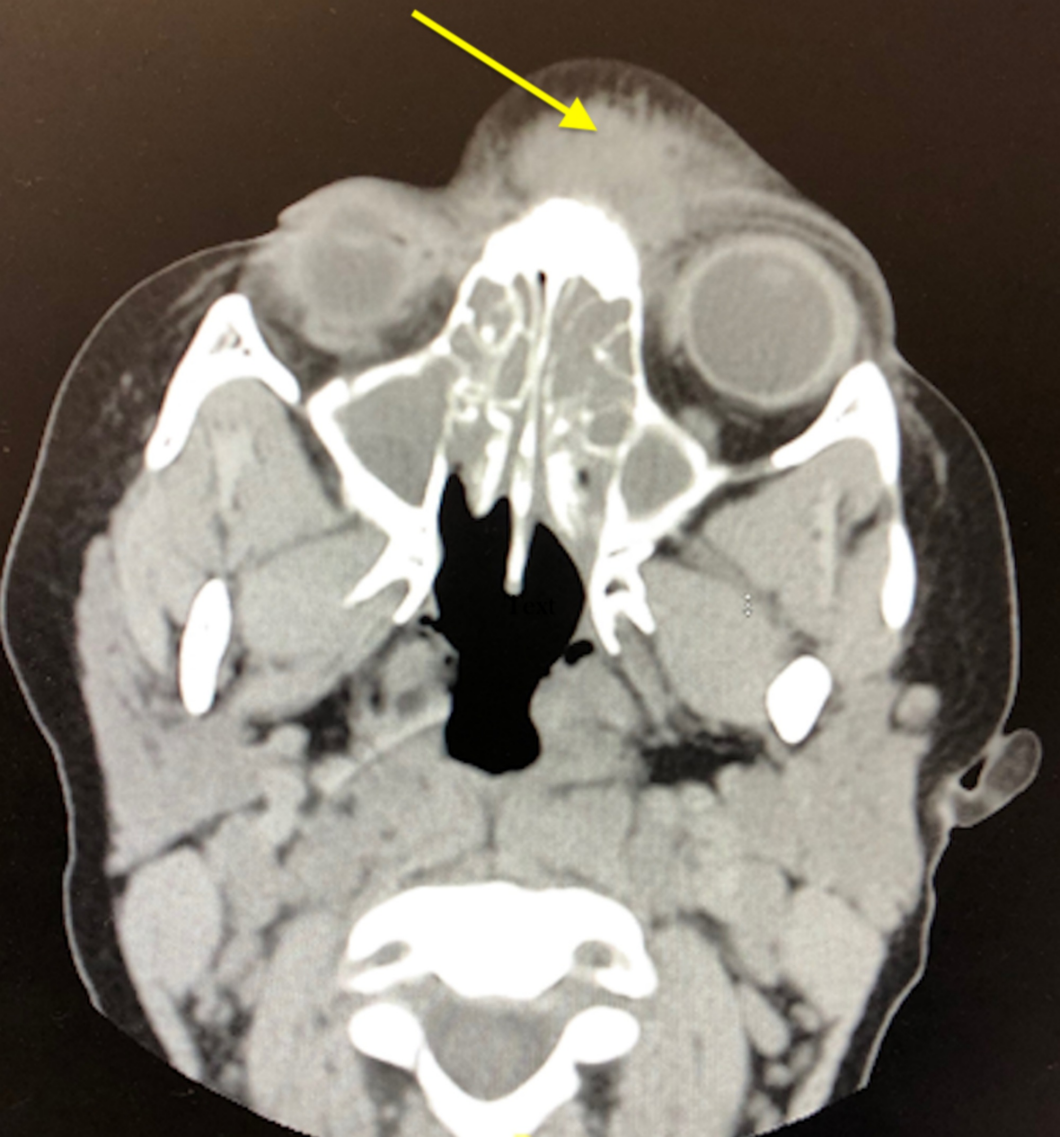
CT scan demonstrating pansinusitis and a large cystic forehead mass (arrow).

CT scan showed fluid accumulation in all the patient’s sinuses. In addition, there was a mass located between the orbits on the forehead which was the result of the pansinusitis (arrow). The radiology impression was read as severe pansinusitis, with inflammatory change through the left frontal sinus and frontal bone into the subcutaneous tissues over the superior nasal region and forehead (Figure 1).

The patient was already taking weight based suspension TMP-SMX every 12 hours and prednisolone once daily. After the CT scan, the patient was diagnosed with pansinusitis and was switched to suspension amoxicillin-clavulanate 10 mL every 12 hours. The patient was discharged home after seeing the otorhinolaryngology consult in the ED.

## Discussion

**Figure 2:**
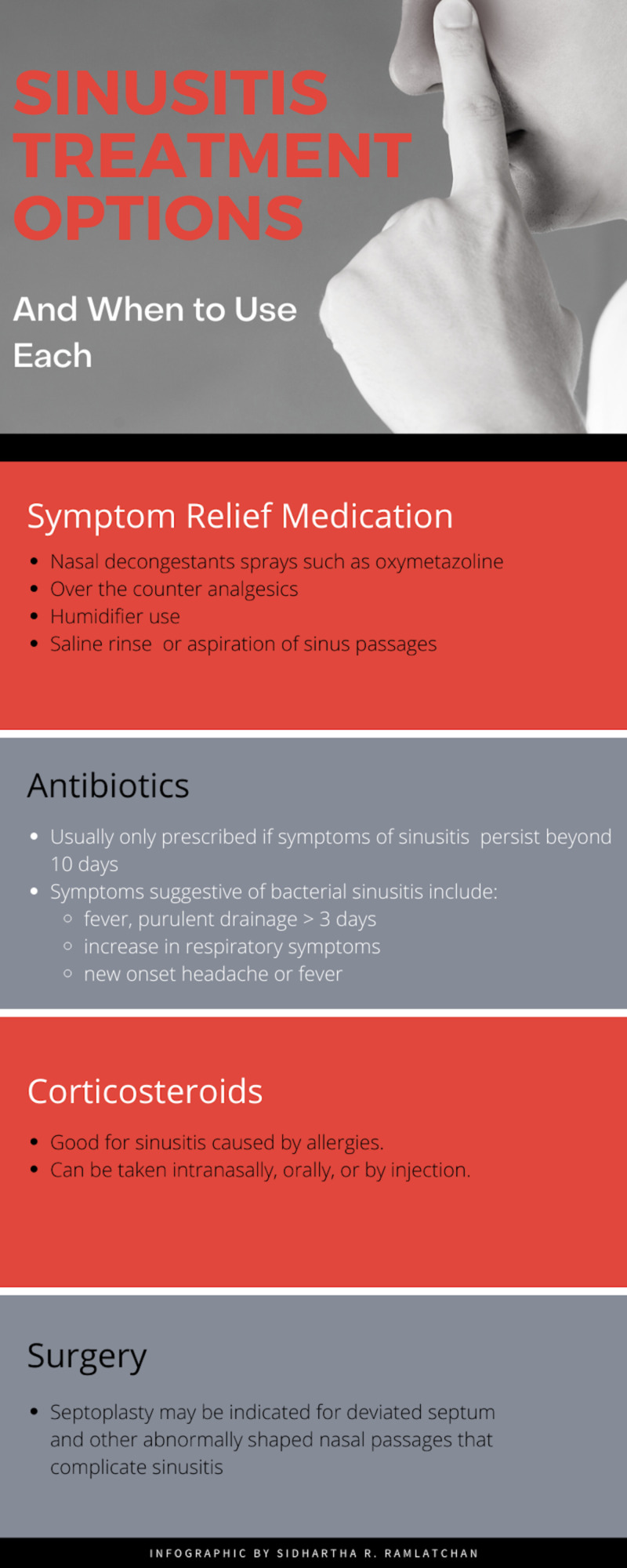
Sinusitis treatment options

The ideal treatment for sinusitis depends on the etiology for the inflammation. In most cases, the etiology of the inflammation is secondary to the common cold from a viral infection. If this is the case, the sinusitis usually tends to cease on its own after about 10 days [3]. Patients can choose to alleviate their symptoms by using over the counter medications for analgesia including acetaminophen or non-steroidal anti-inflammatory drugs (NSAIDs), as well as a nasal decongestant, rest, and humidification. Sinusitis can also be caused by bacteria infection, allergies, chemical or particulate irritation of the sinuses, and mechanical obstruction from deviated septum and nasal polyps [2]. Suspect bacteria causes if symptoms persist greater than 10 days, the condition worsens despite supportive treatment, and the nasal congestion turns mucoid. The below graphic summarizes the treatment options for acute sinusitis (Figure 2).

Bacteria can exist in the pools of mucus that fill the sinuses of a patient with sinusitis. This is a possible complication that arises after inflammation causes mucus to heavily accumulate [5]. Suspect bacteria causes if symptoms persist greater than 10 days, the condition worsens despite supportive treatment, and the congestion turns mucoid. Samples can be taken from a patient’s nasal passage for a bacteria culture test to determine if an acute bacterial sinusitis is present. However, this is not commonly done, especially in the ED setting, and diagnosis is based on clinical suspicion. Antibiotics are recommended for bacterial sinusitis [4].

Cases of sinusitis that persist after 10 days can also be caused by seasonal allergies in which case intranasal corticosteroids are the recommended treatment [6]. These may be in the form of nasal sprays or mists, and aim to reduce irritation and inflammation of the sinuses. Oral corticosteroids can be taken if the intranasal forms deem ineffective, but their use should be limited due to their side effects (including swelling of the lower legs, high blood pressure, and weight gain among others). Injected corticosteroids are also an option, but rarely used.

Abnormalities in bone or cartilage structure may predispose patients to sinusitis and worsen the effects of sinusitis. A deviated septum is a nasal passage abnormality that predisposes to sinusitis due to nasal obstruction that requires definitive correction through surgery called “septoplasty”. Septoplasty straightens a patient’s septum and alleviates the effects of a patient’s sinusitis by removing pieces of bone or cartilage that forces the septum to deviate from the normal position and contribute to nasal obstruction [7]. Nasal polyps are a complication of sinusitis that result from severe inflammation of the sinuses. They are soft tissue masses that extend from nasal tissue and can obstruct breathing and worsen the effects of sinusitis. Endoscopic nasal surgery aims to remove particles blocking the nasal passages, and is recommended for cases of sinusitis that have worsened the effects due to nasal polyps [8].

## Conclusions

Sinusitis can initially present as a simple URI. While many cases of sinusitis will resolve with nasal decongestants and supportive care, some will evolve into a bacterial infection or extend to more than one sinus. When a patient returns with symptoms that have not improved after 10 days, consider that the "cold" or "viral illness" could be a sinusitis that requires further workup or treatment.
